# Chijabyukpi-Tang Inhibits Pro-Inflammatory Cytokines and Chemokines *via* the Nrf2/HO-1 Signaling Pathway in TNF-α/IFN-γ-Stimulated HaCaT Cells and Ameliorates 2,4-Dinitrochlorobenzene-Induced Atopic Dermatitis-Like Skin Lesions in Mice

**DOI:** 10.3389/fphar.2020.01018

**Published:** 2020-07-07

**Authors:** Ji-Hyun Lee, Ji-Ye Lim, Eun Hee Jo, Hyeon Min Noh, Sunggu Park, Min Cheol Park, Dae-Ki Kim

**Affiliations:** ^1^ Department of Immunology and Institute of Medical Sciences, Medical School, Chonbuk National University, Jeonju, South Korea; ^2^ Research Center of Traditional Korean Medicine, Wonkwang University, Iksan, South Korea; ^3^ Department of Acupuncture and Moxibustion, College of Korean Medicine, Wonkwang University, Iksan, South Korea; ^4^ Korean Traditional Medicine Institute, Wonkwang University, Iksan, South Korea; ^5^ Department of Korean Medical Ophthalmology & Otolaryngology & Dermatology, College of Korean Medicine, Wonkwang University, Iksan, South Korea

**Keywords:** *Chijabyukpi-tang*, atopic dermatitis, keratinocytes, cytokine, chemokine, inflammation

## Abstract

*Chijabyukpi-tang* (CBT) is an oriental herbal formula consisting of three herbs (Gardeniae Fructus (*Gardenia jasminoides* J.Ellis.), Phellodendri Cortex (*Phellodendron amurense* Rupr.), Glycyrrhizae Radix (*Glycyrrhiza uralensis* Fisch. ex DC.) at the ratio of 2: 2: 1. CBT has traditionally been used to treat eczema with inflammation in Northeast Asia. The components of CBT have been shown to have anti-inflammatory and anti-oxidant properties, but the exact role and mechanism of CBT on atopic dermatitis (AD) remain unclear. In this study, we investigated the anti-inflammatory effect and mechanism of CBT in the HaCaT human keratinocyte cell line and investigated the anti-atopic effect in mice models of atopic dermatitis-like skin lesions. In the tumor necrosis factor alpha (TNF)-α/interferon (IFN)-γ-stimulated HaCaT cells, CBT inhibited the production of pro-inflammatory cytokines and chemokines and elevated the nuclear translocation of NF-E2 p45 related factors 2 (Nrf2) and subsequent production of heme oxygenase-1 (HO-1). CBT improved the symptoms of atopic dermatitis-like lesions in 2,4-dinitrochlorobenzene (DNCB)-treated mice by suppressing the levels of serum immunoglobulin E (IgE), and various pro-inflammatory cytokines and chemokines. The improvement effect of CBT on atopic dermatitis-like lesions can be predicted to be due to increased Nrf2 and HO-1 gene expression. These results suggest that CBT is an herbal medicine with the potential for use as a therapeutic agent for inflammatory skin diseases such as atopic dermatitis.

## Introduction

Atopic dermatitis (AD) is a chronic relapsing inflammatory skin disease, and the incidence of AD worldwide has been increasing rapidly over the past 30 years (Kapoor et al., 2008). AD occurs most often during childhood and occurs equally in both females and males. In developed countries, about 2–4% of adults and 15–20% of children suffer from AD, and most AD patients are known to have symptoms that last approximately five years (Shaw et al., 2011). AD is characterized by severe skin lesions such as pruritus, erythema, rash, edema, dryness, and skin hypersensitivity (Guttman-Yassky et al., 2011). Because of these features, AD patients often suffer from sleep deprivation, anxiety, and stress, which can lower their quality of life (Kiebert et al., 2002). Many sufferers of AD are also prone to developing other chronic inflammatory diseases including asthma and allergic reactions.

AD is caused by a complex interaction between extrinsic and intrinsic factors. The main risk factor of AD is not yet known, but it is known that mostly immune system dysfunction and environmental factors impair the skin’s barrier and exacerbate immunoglobulin E (IgE)-mediated sensitization, severe skin inflammation and immune responses (Darlenski et al., 2014).

AD skin inflammation is orchestrated by the production of pro-inflammatory cytokines and chemokines by keratinocytes that subsequently induces the invasion of immune cells. The immune cells create inflammatory skin lesions following the production of further pro-inflammatory cytokines and chemokines (Homey et al., 2006). According to previous studies, AD patients are known to have increased levels of chemokines (such as IL-8, CCL17, and CXCL10) and cytokines (such as IL-4, IL-6, and IL-13), compared to healthy individuals (Yamanaka and Mizutani, 2011). It is also known that the activation of several oxidative stress-related inflammatory signaling pathways, such as NF-E2 p45 related factors 2 (Nrf2) and heme oxygenase-1 (HO-1), is related to inflammation in AD (Choi et al., 2014; Akram et al., 2016).

Currently, topical ointments and oral medications such as corticosteroids, antihistamines, and immunosuppressive drugs are used to treat AD inflammation and reduce itching (Cury Martins et al., 2015). However, these drugs have serious side effects when repeatedly administered over a long period of time. Therefore, in order to minimize the adverse effects of the long-term use of AD drugs, there is a need for the development of new alternative drugs derived from natural resources with fewer side effects.

*Chijabyukpi-tang* (CBT) is an oriental herbal formula consisting of three herbs (Gardeniae Fructus, Phellodendri Cortex, Glycyrrhizae Radix), in a 2:2:1 ratio. CBT is a traditional medicine first reported thousands of years ago in the ancient Chinese medicine book “Sang han-lun”. CBT has long been used as a treatment for eczema with inflammation (Mie et al., 2009). In addition, it is an oriental herbal formula that is known to eliminate heat in the body, cure humid, jaundice and epidemic diseases. It has also been used to treat severe pain in the lower abdomen, burns, and various infectious symptoms (Chen et al., 2009). The various effects of each herb that constitutes CBT are well known; previous studies have shown that Gardeniae Fructus and Phellodendri Cortex have anti-inflammatory and anti-allergic effects (Hon et al., 2011; Debnath et al., 2018). In addition, Glycyrrhizae Radix has been previously shown to relieve inflammation (Yang et al., 2013). However, there is few scientific research on the CBT in which these herbs are combined. Previous studies have reported that CBT relieves fever and dizziness, and pruritic skin disease (Higashi and Kanzaki, 2006; Choi and Lee, 2017).

Since AD is characterized by inflammation and pruritus, eczema, we first studied the effect of CBT on AD and its mechanism of action. Therefore, the aim of this study was to investigate the anti-inflammatory effect and action mechanism of CBT on TNF-α/IFN-γ-stimulated HaCaT cells (human keratinocyte cell line) and 2,4-dinitrochlorobenzene (DNCB)-induced AD-like skin lesions in mice.

## Materials and Methods

### Cell Culture and Reagents

The HaCaT cell line was obtained from the laboratory of Wonkwang university (Prof. Min Cheol Park). The HaCaT cells were incubated in Dulbecco’s modified Eagle’s medium (DMEM) supplemented with 10% fetal bovine serum (FBS), 100 units/ml of penicillin, and streptomycin (Welgene, Seoul, Korea). The HaCaT cells were cultured at 37°C in an incubator with a humidified atmosphere of 5% CO_2_ and 95% air. DMEM and FBS were purchased from GIBCO BRL (Grand Island, NY, USA). Penicillin and streptomycin were purchased from Welgene (Seoul, Korea). Recombinant human TNF-α, IFN-γ, and IgE mouse ELISA kit were obtained from BioLegend (San Diego, CA, USA). Primary antibodies for Nrf2, Lamin B, HO-1, β-actin, and secondary antibodies used in the western blot analysis were purchased from Santa Cruz Biotechnology (Santa Cruz, CA, USA). 3-(4,5-Dimethyl-2-thiazolyl)-2,5-diphenyl-2H-tetrazolium bromide (MTT), dexamethasone (#D2915), and DNCB were purchased from Sigma-Aldrich. (St. Louis, Mo., USA).

### Preparation of CBT

The herb components used in CBT were obtained from the K-herb Research Center, Nong-Lim and Ja-Dam. The identity of the herbs was confirmed by Prof. Min-Chel Park from Wonkwang University of traditional korean medicine. Voucher specimens of Gardeniae Fructus (NLGF-1803) and Phellodendri Cortex (NLPC-1801) have been deposited at Nong-Lim. Voucher specimen of Glycyrrhizae Radix (JD8KZ-1902) has been deposited at Ja-Dam. CBT was constructed by mixing the herbs in accordance with each component composition in **Table 1** (www.theplantlist.org). 18 g of CBT was boiled at 100°C in 1,000 ml water for 30 min. Then the extract was collected, and as above, the residue was twice boiled in water. The extracted CBT was filtered and freeze-dried, and the weight of the dried extract was measured, and kept at 4°C until use.

**Table 1:** The composition of *Chijabyukpi-tang* (CBT).

Herb medicine	Latin scientific name	Family	Source	Weight (g)/Ratio (%)
**Gardeniae Fructus**	*Gardenia jasminoides* J.Ellis.	Rubiaceae	Korea	6/40
**Phellodendri Cortex**	*Phellodendron amurense* Rupr.	Rutaceae	Korea	6/40
**Glycyrrhizae Radix**	*Glycyrrhiza uralensis* Fisch. ex DC.	Fabaceae	Korea	3/20
**Total Amount**				**15/100**

### Analysis of High Performance Liquid Chromatography (HPLC)

To perform the HPLC analysis of CBT, the Waters e2695 separation module, 2998 photodiode array (PDA) detector (Waters Corporation, USA) was used. The analytical column used was Phenomenex Luna C18 (250× 4.6 mm). The column temperature was at 40°C, the samples temperature was at 25°C, and the injection volume was 10 μl. The mobile phases were composed of solvent (A) = 0.1% trifluoroacetic acid in water, and solvent (B) = Acetonitrile. The total run time was 120 min and the mobile phase process gradient flow was as follows: (A)/(B) = 80/20 (0-10 min) → (A)/(B) = 80/20 (10-90 min) → (A)/(B) = 40/60 (90-91 min) → (A)/(B) = 0/100 (91-111 min) → (A)/(B) = 80/20 (111-120 min). The mobile phase flow rate was 1.0 ml/min. Samples were detected with a UV detector at a wavelength of 260 nm.

### MTT Assay

The viability of HaCaT cells was evaluated using the MTT assay. The MTT assay was carried out as previously described (Lee et al., 2018). Briefly, cells were seeded into a 96-well plate at 1 × 10^4^ cells/well, and treated with various concentrations of CBT (0, 12.5, 25, 50, 100 µg/ml) for 24 h in a 37°C incubator. MTT reagents were added to each well, and the plate was incubated for a further 4 h. After removing the supernatant, the crystallized formazan was dissolved in dimethyl sulfoxide (DMSO), and the absorbance was read at 570 nm using an ELISA plate reader.

### Preparation of the HaCaT Nuclear and Cytosol Fractions

The nuclear and cytosol fractions of the HaCaT cells were isolated using the Nuclear/Cytosol Fractionation Kit (BioVision, Inc., CA, USA), according to the manufacturer’s instructions. The nuclear and cytosol fractions were stored at -80°C until use.

### Quantitative Real-Time Polymerase Chain Reaction (qPCR)

qPCR was carried out to confirm the expression levels of several genes *in vitro* and *in vivo*. Total RNA was isolated from *dorsal skin tissues* and HaCaT cells using 1 ml Trizol *reagent* (Invitrogen, Carlsbad, CA, USA). After the RNA was extracted, cDNA was synthesized using the Prime Script ™ II 1st strand cDNA synthesis kit (Takara Bio, Inc. USA), according to the *manufacturer’s protocols.* The cDNA was amplified with SYBR Green PCR Master Mix (Applied Biosystem, CA, USA) using an ABI Real-Time PCR system (Applied Biosystems, Inc., CA, USA). *The primer sequences are shown in* **Table 2**. The RNA gene expression levels of each sample were analyzed three times and normalized to the internal control gene, GAPDH.

**Table 2:** *Primer sequences for* quantitative real-time polymerase chain reaction (qPCR).

Gene	Forward	Reverse
hIL-6	CTCCACAAGCGCCTTCGGTC	TGTGTGGGGCGGCTACATCT
hIL-13	ACCACGGTCATTGCTCTCACT	GTCAGGTTGATGCTCCATAC
hIL-8	ACCGGAGCACTCCATAAGGCA	AGGCTGCCAAGAGAGCCACG
hCCL17	CCATTCCCCTTAGAAAGCTG	CTCTCAAGGCTTTGCAGGTA
hCXCL10	TTGCTGCCTTATCTTTCTGACTC	ATGGCCTTCGATTCTGGATT
mIL-4	ATGGGTCTCAACCCCCAGCTA	TGCATGGCGTCCCTTCTCCT
mIL-6	GACAACCACGGCCTTCCCTA	GGTACTCCAGAAGACCAGAGG
mIL-8	TTTGGGAGACCTGAGAACAAG	TGCCTGTCAAGCTGACTTCA
mCXCL10	CTGAGTGGGACTCAAGGGAT	TCGTGGCAATGATCTCAACAC
mHO-1	CAGAACCAGCCTGAACTAGC	TGGATGTGTACCTCCTTGGT
mNrf2	ACCAAGGGGCAC CATATAAAAG	CTTCGCCGAGTTGCACTCA
GAPDH	GAAGGTGAAGGTCGGAGT	GAAGATGGTGATGGGATTTC

IL-, Interleukin-; CCL17, Chemokine (C-C motif) ligand 17; CXCL10, C-X-C motif chemokine 10; HO-1, heme oxygenase-1; Nrf2, NF-E2 p45-related factor 2; GAPDH, glyceraldehyde 3-phosphate dehydrogenase.

### Western Blot Analysis

The protein production levels of cells were evaluated using western blot analysis. HaCaT cells were pretreated with various concentrations of CBT (0, 12.5, 25, 50, and 100 μg/ml) for 2 h, and treated with TNF-α/IFN-γ (10 ng/ml each) for 3 h. The cells were harvested, and proteins were extracted using cell lysis buffer (Millipore, Bedford, MA, USA) supplemented with a protease and phosphatase inhibitor cocktail (Thermo Fisher Scientific, Waltham, MA, USA). Proteins (25 μg) were separated *via* electrophoresis in 10% sodium dodecyl sulfate-polyacrylamide (SDS-PAGE) gels, and transferred to PVDF membranes. Membranes were blocked with 5% BSA in TBS-T at room temperature for 1 h, and incubated with primary antibodies (Nrf2, Lamin B, HO-1, and β-actin; 1:1,000 dilution in 5% BSA) overnight at 4°C. Then, the membranes were washed several times with TBS-T, and incubated with horseradish peroxidase (HRP)-conjugated secondary antibody (1:5,000 dilution in 5% BSA) at room temperature for 1 h. The proteins were detected with enhanced chemi-luminescence (ECL) detection kit (Millipore, Billerica, MA, USA) using the Fusion Fx gel documentation system (Davinch-Invivo™ Imaging System, USA).

### Animals

Four-week-old male BALB/c mice were purchased from Samtako Bio Korea (Osan, Korea). All mice (22 ± 2 g) were randomly housed in a controlled environment with 50–60% humidity and a temperature of 21–23°C, under a 12-h dark/light cycle. Mice were allowed free access to standard laboratory chow and tap water. After a one-week period of new environment acclimation, mice were randomly divided into five groups of six mice. This research was performed in accordance with the guidelines of the Animal Experiment Ethics Committee of Chonbuk National University (CBNU-IACUC) (Confirmation No. CBNU 2016-0011).

### Experimental Protocols of the Mouse Study

Thirty mice were divided into five groups (n=6) as follows: (1) control (vehicle treatment), (2) DNCB, (3) DNCB + CBT-low dose (CBT-150 mg/kg), (4) DNCB + CBT-high dose (CBT-300 mg/kg) and (5) positive control (dexamethasone (Dexa)-1 mg/kg). Except for the control group, AD-like skin lesions were induced using DNCB as described previously. Briefly, the dorsal skin hairs of all mice were removed. After 24 h, DNCB was dissolved in an acetone and olive oil mixture (4:1 v/v), and the dorsal skin was treated with 1% DNCB solution once a day for three days. Furthermore, 0.5% DNCB solution was treated to the dorsal skin once every two days for ten days to promote AD-like skin lesions. CBT samples and dexamethasone were dissolved in water and orally administered once a day for 11 days (days 4–14) (**Figure 4A**). The experimental animals were anesthetized by ether and euthanized by cervical dislocation for collecting skin tissues and blood serum.

### Measurement of Dorsal Skin Moisture Content, Atopic Dermatitis Score, and Dorsal Skin Thickness

On the last day of the experiment, the dorsal skin moisture content (%) was measured using a TS-skin diagnostic system (Aram Huvis Co., Seongnam, Korea), according to the manufacturer’s protocol. We assessed the severity of erythema, edema, and stretches on AD-like skin lesions on the dorsal skin five times according to Fan’s criteria (Fan et al., 2018). After the dorsal skin tissues of mice were isolated, the thicknesses of the dorsal skin were gauged three times using a micrometer.

### Hematoxylin and Eosin (H&E) Staining

After the mice were sacrificed, their dorsal skin tissue specimens (100 μm^2^) were fixed in 10% formalin solution at room temperature for 24 h, and then embedded in paraffin wax. The paraffin blocks were sectioned serially into a thickness of 6-μm (n=6), and stained with hematoxylin and eosin (H&E; hematoxylin, 1 min and eosin, 3 min) to examine skin histological changes. Histological changes were observed using a microscope (Olympus CX21, Olympus Corporation, Tokyo, Japan). All images were observed at ×100 magnification.

### Enzyme-Linked Immunosorbent Assay (ELISA)

After the mice were anesthetized, blood samples were collected. Blood samples were allowed to clot by incubation at room temperature for 30 min. Then, bloods were centrifuged at 3,000 rpm for 10 min and serum samples were obtained. IgE in serum was analyzed using a total IgE mouse ELISA kit, according to the manufacturer’s instructions.

### Statistical Analysis

Graph Pad Prism software 5.0 was used for data statistical analysis. All data are represented as means ± standard error of the mean (SEM) of triplicate experiment, and evaluated using one-way ANOVA (analysis of variance) with Tukey’s *post-hoc* test to locate differences between groups. A value of *p* < 0.05 was defined as statistically significant.

## Results

### HPLC Analysis of CBT

**Figure 1:**
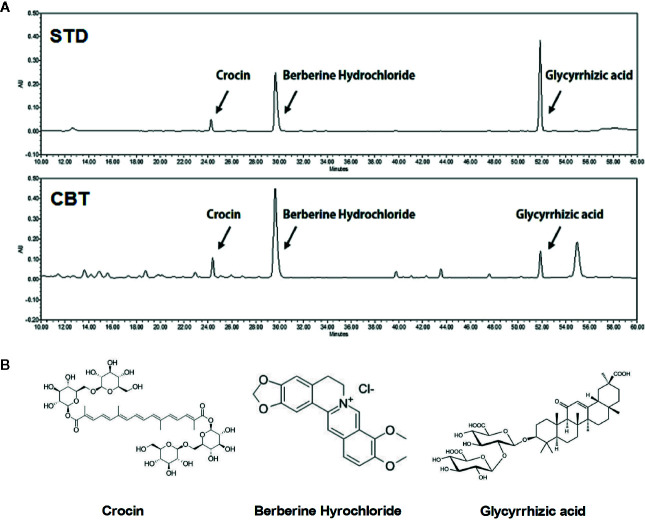
High performance liquid chromatography **(**HPLC) analysis of *Chijabyukpi-tang* (CBT). HPLC chromatograms of standard compound mixtures (STD) and CBT samples **(A)**. Chemical structures of crocin, berberine hydrochloride, and glycyrrhizic acid **(B)**.

The final extraction yield (%) of CBT was 37.77%. The CBT indicator ingredient compounds (crocin, berberine hydrochloride, and glycyrrhizic acid) were analyzed using HPLC. We confirmed the peak of three compounds in the CBT extract by comparison with the standard ingredients’ peak. As shown in **Figure 1**, the CBT used in our experiments contains these three components. The content of crocin, berberine hydrochloride, and glycyrrhizic acid in CBT was 28.671 mg/g, 6.255 mg/g, and 6.249 mg/g, respectively.

### CBT Reduces the mRNA Expression of Pro-Inflammatory Cytokines and Chemokines in TNF-α/IFN-γ-Stimulated HaCaT Cells

**Figure 2:**
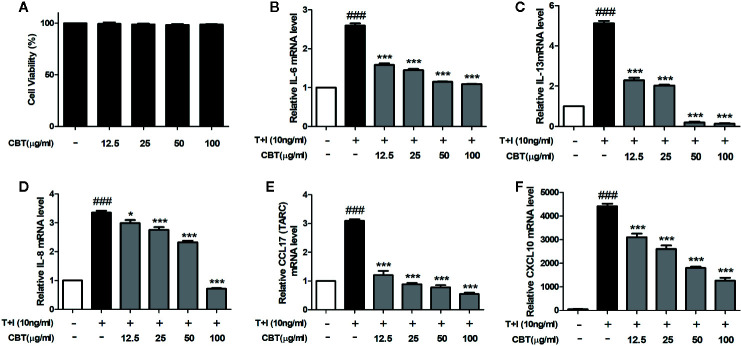
Effects of *Chijabyukpi-tang* (CBT) on the mRNA expression levels of pro-inflammatory cytokines and chemokines in TNF-α/IFN-γ-stimulated HaCaT cells. Cell viability of CBT was measured using MTT assay. The group that did not treatment anything was set as negative control **(A)**. Cells were incubated for 24 h in the presence of CBT (12.5 to 100 μg/ml) or in the absence of CBT (media only). HaCaT cells were pretreated with several concentrations of CBT (12.5, 25, 50, and 100 μg/ml) for 2 h and then stimulated with TNF-α/IFN-γ (each 10 ng/ml) for 3 h. The mRNA expression levels of IL-6 **(B)**, IL-13 **(C)**, IL-8 **(D)**, CCL17 **(E)**, and CXCL10 **(F)** were determined using quantitative real-time polymerase chain reaction (qPCR). Data are shown as mean ± SEM of the three independent experiments. *^###^p <* 0.001 compared with the no-treatment condition, ^*^p < 0.05, and ^***^p < 0.001 compared with the only TNF-α/IFN-γ treatment condition.

The cytotoxicity of HaCaT cells was examined at various concentrations (12.5, 25, 50, and 100 μg/ml) of CBT for 24 h. As shown in **Figure 2A**, CBT did not show cytotoxicity at all concentration ranges. To evaluate the anti-inflammatory effects of CBT, the production of pro-inflammatory cytokines and chemokines was measured in TNF-α/IFN-γ-treated HaCaT cells. HaCaT cells were pretreated with CBT (0, 12.5, 25, 50, and 100 μg/ml) for 2 h, and stimulated with TNF-α/IFN-γ (10 ng/ml each) for 3 h. Then, the mRNA expression levels of cytokines (IL-6 and IL-13), and chemokines (IL-8, CCL17, and CXCL10) were analyzed using qPCR. CBT was shown to significantly inhibit the production of TNF-α/IFN-γ-induced cytokines and chemokines in a dose-dependent manner (**Figures 2B–F**).

### CBT Up-Regulates Nuclear Nrf2 and HO-1 Protein Levels in TNF-α/IFN-γ-Stimulated HaCaT Cells

**Figure 3:**
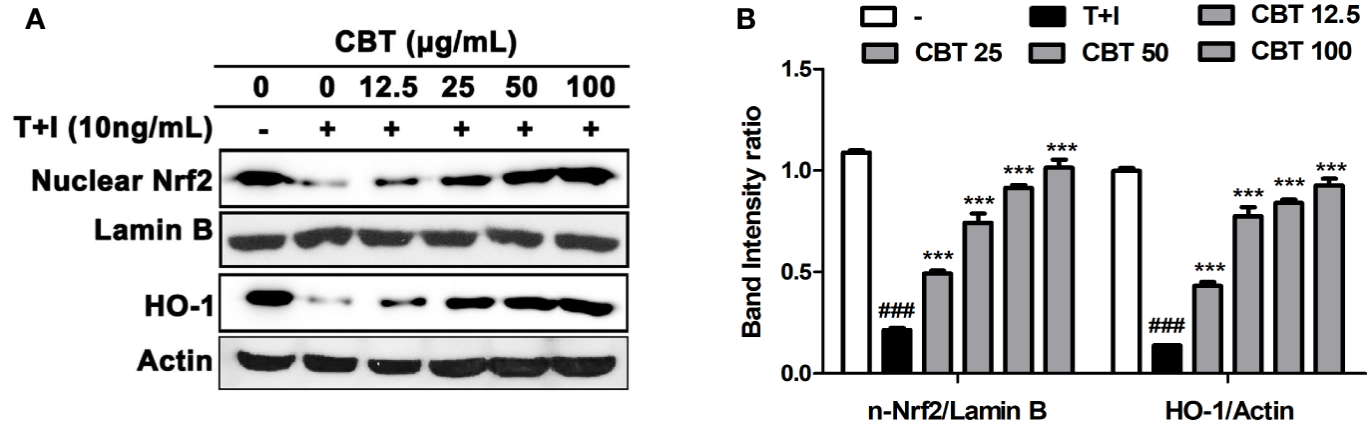
Effects of *Chijabyukpi-tang* (CBT) on the nuclear Nrf2 and HO-1 proteins in TNF-α/IFN-γ-stimulated HaCaT cells. HaCaT cells were pretreated with several concentrations of CBT (12.5, 25, 50, and 100 μg/ml) for 2 h and then stimulated with TNF-α/IFN-γ (each 10 ng/ml) for 3 h. The proteins of nuclear Nrf2, lamin B, HO-1, and actin **(A)** were analyzed using western blotting. The bar graphs represent quantitative density of the bands; nuclear Nrf2/Lamin B, and HO-1/Actin **(B)**. Data are shown as mean ± SEM of the three independent experiments. *^###^p <* 0.001 compared with the no-treatment condition, ^***^p < 0.001 compared with the only TNF-α/IFN-γ treatment condition.

The Nrf2/HO-1 signaling pathway plays a crucial mediator role in cell protection from oxidative stress (Loboda et al., 2016). To evaluate whether CBT affects the Nrf2/HO-1 signaling pathway, we investigated the levels of nuclear Nrf2 and HO-1 proteins using western blot. As shown in **Figure 3**, protein levels of nuclear Nrf2 and HO-1 were decreased following cellular stimulation with TNF-α/IFN-γ, however in the groups pretreated with CBT, the nuclear Nrf2 and HO-1 activation was increased in a dose-dependent manner.

### CBT Improves DNCB-Induced AD-Like Skin Lesions and Suppresses the Secretion of Serum IgE in Mice

**Figure 4:**
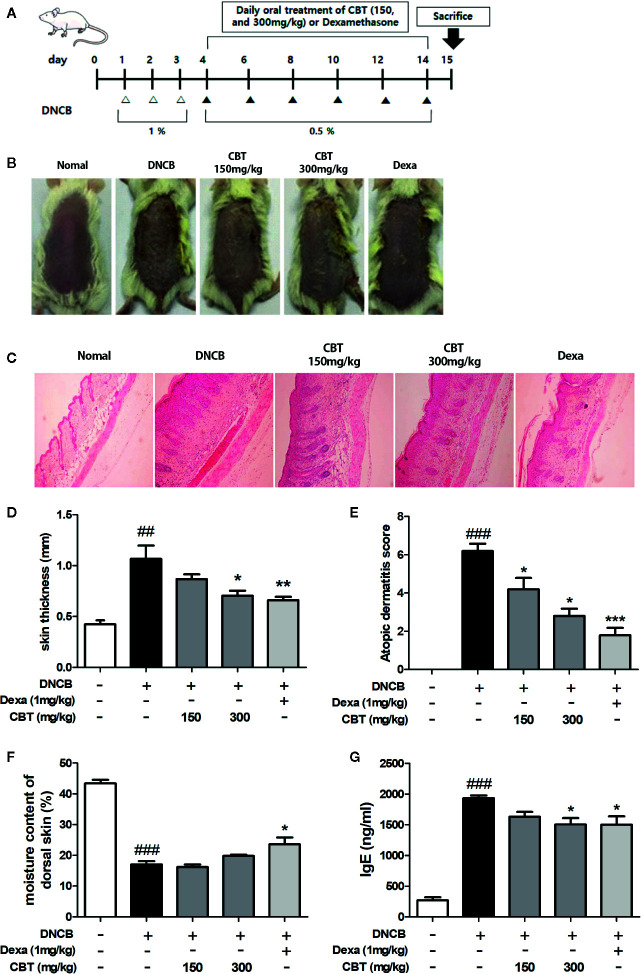
Effects of *Chijabyukpi-tang* (CBT) on clinical signs in 2,4-dinitrochlorobenzene (DNCB)-treated mice skin tissues. The DNCB treatment and oral administration (CBT 150 and 300 mg/kg, and Dexa) schedule used in this experiment **(A)**. On the last day of the experiment, photographs were taken for comparison of skin lesions before the mice were sacrificed. Each dorsal skin picture is representative of each group **(B)**. The histological status of the dorsal skin tissues was observed using hematoxylin and eosin (H&E) staining **(C)**. Images were taken at 100x magnification. The dorsal skin tissue thickness of mice was measured using a micrometer **(D)**. The atopic dermatitis score of mouse dorsal skin lesion was evaluated using the standard evaluation criteria **(E)**. The moisture content of mouse dorsal skin was measured using a Tewameter TM 210 device **(F)**. Serum IgE level was analyzed using enzyme-linked immunosorbent assay (ELISA) **(G)**. Data are shown as mean ± SEM of the three independent experiments. *^###^p <* 0.001 compared with the control group, ^*^p < 0.05, ^**^p < 0.01, and ^***^p < 0.001 compared with the DNCB-induced group. The Dexa group was set as positive control.

AD-like skin lesions were induced by repeated exposure of the dorsal skin regions of BALB/c mice to DNCB. A significant improvement in dorsal skin condition was observed in mice administrated CBT orally compared to the only DNCB-induced group (n=6) (**Figure 4B**). There were no mouse deaths by CBT (150 and 300 mg/kg) oral administration. We examined H&E staining slides to study the therapeutic effects of CBT on epidermal hyperplasia and the infiltration of inflammatory cells in AD-like dorsal skin tissue. As a result, we found that the dorsal skin thickness of DNCB-induced mice was increased, and that oral administration of CBT reduced the dorsal skin thickness and relieved AD-like lesions in a dose dependent manner (**Figures 4C, D**). The severity scores of the dorsal skin lesions were evaluated with reference to known criteria. We observed that AD-severity scores were suppressed in the CBT-orally administrated mice groups in a dose-dependent manner (**Figure 4E**). We observed a significant decrease in dorsal skin moisture content (%) of approximately 60.9% in the DNCB-treated mice group compared to the non-DNCB-treated mice group. When orally administered with CBT, the DNCB-treated mice group’s skin moisture content was elevated. However, no significant difference was observed (**Figure 4F**). IgE is associated with the T helper 2 (Th2) immune response and is known to play an important role in the pathogenesis and progression of AD (Liu et al., 2011). Thus, total IgE measurements have been used to confirm the severity of AD. We analyzed the effect of CBT on serum IgE levels using ELISA. As a result, serum total IgE was increased in the DNCB-treated mice group compared with the control mice group (control mice group: 267.81 ± 84.81 ng/ml; DNCB-treated mice group: 1,932.53 ± 79.57 ng/ml), and it was reduced by oral administration of CBT (CBT 300 mg/kg administered group: 1,503.92 ± 181.21 ng/ml). In particular, the CBT 300 mg/kg and dexamethasone 1 mg/kg mice groups were significantly inhibited (dexamethasone administered group: 1,500.47 ± 236.26 ng/ml) (**Figure 4G**).

### CBT Inhibits the mRNA Expression of Pro-Inflammatory Cytokines and Chemokines in DNCB-Treated Mice

**Figure 5:**
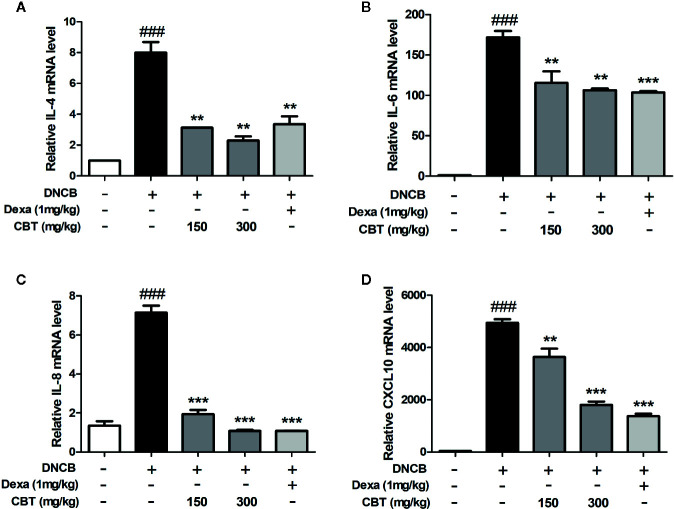
Effects of *Chijabyukpi-tang* (CBT) on the mRNA expression levels of pro-inflammatory cytokines and chemokines in 2,4-dinitrochlorobenzene (DNCB)-treated mice skin tissues. The mRNA expression levels of IL-4 **(A)**, IL-6 **(B)**, IL-8 **(C)**, and CXCL10 **(D)** were analyzed using the quantitative real-time polymerase chain reaction (qPCR). Data are shown as mean ± SEM of the three independent experiments. *^###^p <* 0.001 compared with the control group, ^**^p < 0.01, and ^***^p < 0.001 compared with the DNCB-induced group. The Dexa group was set as positive control.

Dorsal skin tissues were collected from each mouse, and the effect of CBT on pro-inflammatory cytokines and chemokines was analyzed using qPCR. In dorsal skin tissues, the expression levels of pro-inflammatory cytokines (IL-4 and IL-6), and chemokines (IL-8 and CXCL10) were remarkably increased in the DNCB-treated mice group compared with the control mice group. Expression levels of pro-inflammatory cytokines and chemokines in the CBT-orally administered mice groups were reduced compared to the DNCB-treated mice group in a dose-dependent manner (**Figure 5**).

### CBT Elevates the mRNA Expression of Nrf2 and HO-1 in DNCB-Treated Mice

**Figure 6:**
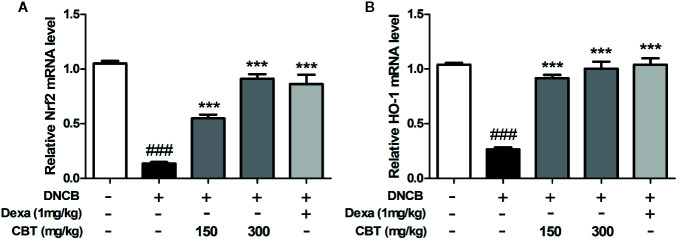
Effects of *Chijabyukpi-tang* (CBT) on the mRNA expression level of HO-1 in DNCB-treated mice skin tissues. The mRNA expression levels of Nrf2 **(A)** and HO-1 **(B)** were analyzed using the quantitative real-time polymerase chain reaction (qPCR). Data are shown as mean ± SEM of the three independent experiments. *^###^p <* 0.001 compared with the normal group, ^***^p < 0.001 compared with the DNCB-treated group. The Dexa group was set as positive control.

The mRNA expression levels of Nrf2 and HO-1 were measured using qPCR. We observed that the mRNA expression levels of Nrf2 and HO-1 decreased by treatment with DNCB were increased by oral administration of CBT in a dose-dependent manner (**Figure 6**).

## Discussion

AD is a chronic inflammatory skin disease that occurs frequently in infancy and has a high rate of both recurrence and persistence. AD is characterized by severe itching, eczema, erythema, dry skin, and skin hyperplasia, and is also closely related to other inflammatory diseases (Peng and Novak, 2015). In inflammatory AD skin lesions, the levels of serum IgE, cytokines and chemokines are increased, and there is infiltration of Th cells (Leung and Bieber, 2003). Although these factors causing AD progression have been outlined, the exact causes of AD and treatments without side effects have yet to be clarified (Dupuy, 1994). Therefore, there is a need to develop safe and effective therapies for AD, using natural extracts and herbal medicines.

For thousands of years, Northeast Asia (Korea, China, and Japan) has used traditional medicinal herb formulas to treat many diseases. However, since there is no scientific evidence for the efficacy and safety of these medicinal herb formulas, consumers have doubts concerning their use. CBT has been used as a treatment for eczema accompanied with inflammation, but there is still no definitive evidence for the efficacy of CBT treatment on AD. Therefore, we conducted this study to investigate the anti-inflammatory effects of CBT on AD and its mechanism of action.

Keratinocytes are the main cells that compose the stratum corneum and play a key role in AD pathogenesis. When keratinocytes are damaged by repetitive mechanical stimulations such as scratching behavior, various keratinocyte-derived cytokines and chemokines are secreted to promote inflammatory skin disease progression (Asahina and Maeda, 2017). These secreted factors also cause additional responses by recruiting immune cells such as neutrophils, monocytes, and T cells to AD inflammatory skin lesions (Giustizieri et al., 2001). T cells recruited to AD inflammatory skin lesions secrete various cytokines such as IL-4, IL-5, IL-6, and IL-13, and chemokines such as IL-8, CCL17, and CXCL10 (Leung and Sorter, 2001). This secretion of pro-inflammatory cytokines and chemokines is known to be caused by the activation of various signaling pathways. Oxidative stress is one of the factors that drive the progression of AD. Since anti-oxidant enzymes play a major role in protecting cells from oxidative stress, increasing anti-oxidant enzymes is an important strategy for treating AD. As a defense against oxidative stress in cells, Nrf2, a transcription factor, moves from the cytosol to the nucleus and increases the expression of anti-oxidant enzyme genes including HO-1. The increased level of HO-1 protein induces cell protection (Ahmed et al., 2017). The Nrf2/HO-1 signaling pathway is involved in the recruitment of various inflammatory cells into inflammatory lesions and is known to contribute to the anti-inflammatory process (Chen et al., 2006; Ryter et al., 2006). In addition, previous studies have indicated that increased HO-1 gene expression attenuates the development of AD skin lesions (Kirino et al., 2008).

Based on these mechanisms, we investigated the anti-inflammatory effect of CBT on TNF-α/IFN-γ-stimulated keratinocytes, and on DNCB-induced AD-like skin lesions in mice.

Firstly, the major compounds of CBT were identified by HPLC, and it was confirmed that it contains crocin (28.671 mg/g), berberine hydrochloride (6.255 mg/g), and glycyrrhizic acid (6.249 mg/g). Previous studies have reported these compounds have anti-inflammatory and anti-oxidant effects (Fagot et al., 2018; Wang et al., 2018).

HaCaT cells are used in many skin disease studies as they can mimic the symptoms of AD in response to inflammatory stimuli such as TNF-α/IFN-γ. In this study, we observed that CBT induces no toxicity in HaCaT cells, and significantly inhibits the increase in mRNA levels of pro-inflammatory cytokines and chemokines in TNF-α/IFN-γ-stimulated HaCaT cells. To further understand the mechanisms of the regulation of the immune response by CBT, we investigated the effects of CBT on the Nrf2/HO-1 signaling pathway. As a result, we confirmed that CBT promoted the migration of Nrf2 into the nucleus and increased the expression levels of the HO-1 gene in a dose-dependent manner. These results suggest that CBT has anti-inflammatory and protective effects on cells by inhibiting the production of pro-inflammatory cytokines and chemokines *via* the Nrf2/HO-1 signaling pathway.

To establish the AD experimental animal model, DNCB was repeatedly applied to the hairless dorsal skin of BALB/c mice (Matsumoto et al., 2004). The mice treated with DNCB were found to exhibit general AD symptoms such as increasing skin thickness, keratinization, and skin dryness. CBT attenuated the symptoms of mice with AD-like skin lesions in a dose-dependent manner. CBT improved the external condition of the dorsal skin and reduced its thickness. In addition, it has also been observed that the reduction in the moisture content of dorsal skin induced by DNCB-treatment is reversed by oral administration of CBT. DNCB-induced dorsal skin damage leads to AD-like inflammatory skin disease characterized by secretion of a variety of pro-inflammatory cytokines and chemokines by damaged keratinocytes resulting in Th cell activation. In this experiment, serum IgE levels, and expression levels of various cytokines and chemokines in skin tissues were increased by DNCB stimulation. These increased serum IgE and gene expression levels in skin tissues were significantly inhibited by the oral administration of CBT. These data provide experimental evidence for the anti-atopic effect of CBT in AD animal models. In these animal models, we also observed expression levels of Nrf2 and HO-1 in tissues to investigate whether the anti-inflammatory response was mediated through the Nrf2/HO-1 signaling pathway. As a result, we found that the expression levels of the Nrf2 and HO-1 gene were increased in a dose-dependent manner by CBT, similar to the result seen in the HaCaT cells.

**Figure 7:**
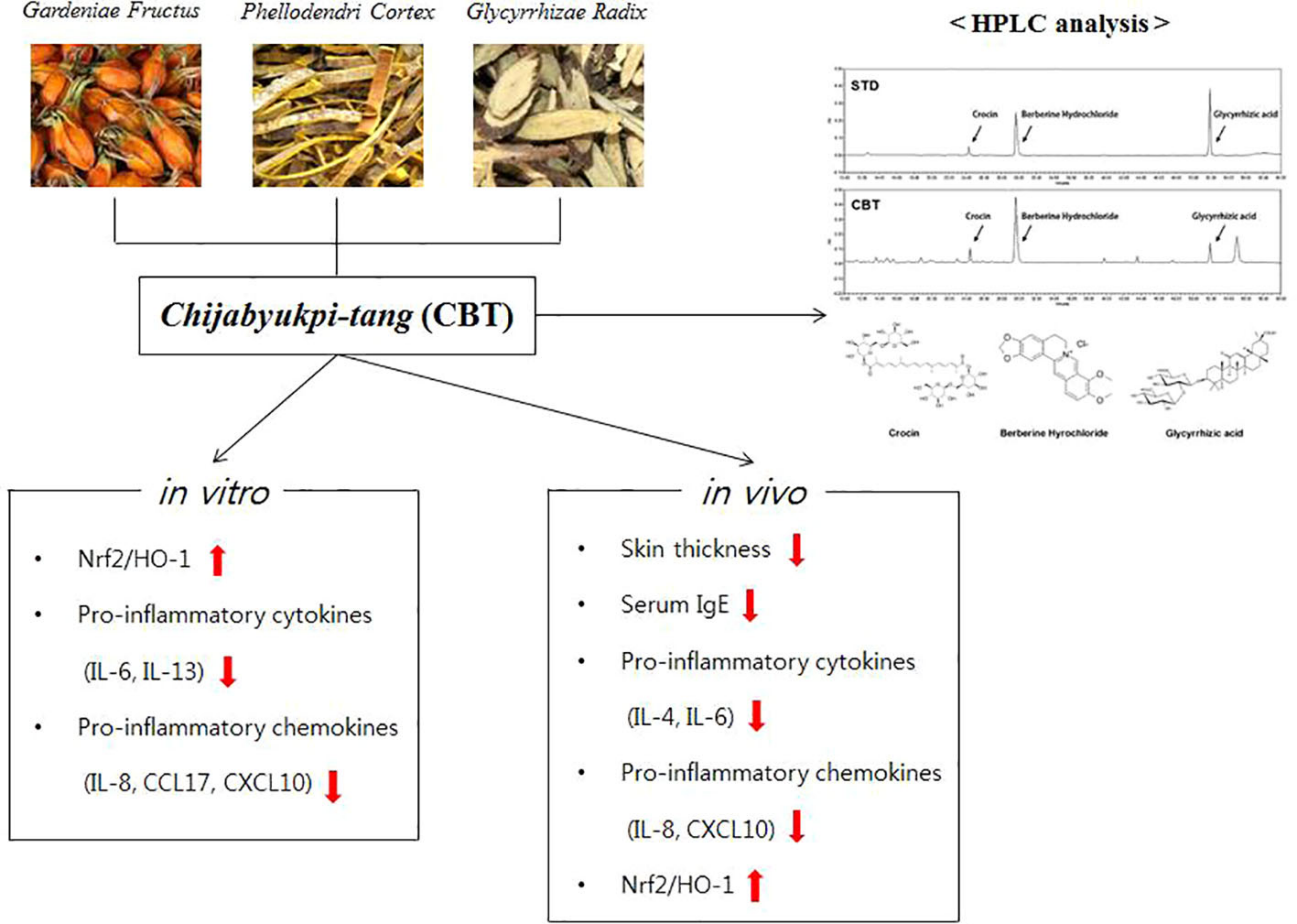
A graphic summary of atopic dermatitis (AD)-like symptoms and related genes regulated by *Chijabyukpi-tang* (CBT).

In conclusion, CBT induces anti-inflammatory effects by up-regulating Nrf2/HO-1 signaling in TNF-α/IFN-γ-stimulated HaCaT cells. In addition, CBT improves AD-like skin lesions and inhibits inflammatory cytokines and chemokines in DNCB-treated mice models (**Figure 7**). These results suggest that CBT is a potential therapeutic candidate for the treatment of AD. However, the effects of dexamethasone or the individual herbal components (crocin, berberine hydrochloride and glycyrrhizic acid) vs CBT are not yet known, and further studies are required.

## Data Availability Statement

All datasets generated for this study are included in the article/supplementary material.

## Ethics Statement

The animal study was reviewed and approved by the Animal Experiment Ethics Committee of Chonbuk National University (CBNU-IACUC) (Confirmation No. CBNU 2016-0011).

## Author Contributions

D-KK, MP, J-HL, and J-YL designed the study. J-HL and J-YL performed the experiments, analyzed the date. J-YL wrote the draft manuscript. J-HL rewrote and revised the manuscript. EJ, HN, and SP read the manuscript. All authors contributed to the article and approved the submitted version.

## Conflict of Interest

The authors declare that the research was conducted in the absence of any commercial or financial relationships that could be construed as a potential conflict of interest.

## Abbreviations

AD, atopic dermatitis; CBT, *Chijabyukpi-tang*; Dexa, dexamethasone; DMSO, dimethyl sulfoxide; DNCB, 2,4-dinitrochlorobenzene; ECL, enhanced chemi-luminescence; ELISA, Enzyme-linked immunosorbent assay; FBS, fetal bovine serum; HO-1, heme oxygenase-1; HPLC, high performance liquid chromatography; H&E, Hematoxylin and eosin; IFN-γ, interferon-γ; IgE, immunoglobulin E; KIOM, Korea Institute of Oriental Medicine; MTT, 3-(4,5-Dimethyl-2-thiazolyl)-2,5-diphenyl-2H-tetrazolium bromide; Nrf2, NF-E2 p45 related factors 2; PDA, photodiode array; qPCR, Quantitative real-time polymerase chain reaction; SDS-PAGE, sodium dodecyl sulfate-polyacrylamide; SEM, standard error of the mean; Th2, T helper 2; TNF-α, tumor necrosis factor-α.
